# Supplementary material for: Gene flow in the Antarctic bivalve Aequiyoldia eightsii (Jay, 1839) suggests a role for the Antarctic Peninsula Coastal Current in larval dispersal
Source: R Soc Open Sci. 2020 Sep 16;7(9):200603. doi: 10.1098/rsos.200603 (PMC7540763; doi:10.1098/rsos.200603)
Supplement: Additional methodological details, figures and tables [file rsos200603supp1.docx]

Electronic Supplementary Material

**Asymmetric gene flow in the Antarctic bivalve *Aequiyoldia eighitsi* suggest a role for the Antarctic Peninsula Coastal Current in larval dispersal**

Muñoz-Ramírez Carlos^1,2,3^, Barnes David KA^4^, Cardenas Leyla^5^, Meredith Mike^4^, Morley Simon A^4^, Roman-Gonzalez Alejandro^6^, Sands Chester J^4^, Scourse James^6^, Brante Antonio^2,3^

**Additional methodological details**

Given the different sensitivities of each analysis to missing data, we produced data sets with three levels of coverage. There is a compromise between number of loci and amount of missing data, where more loci are obtained by allowing higher missing data. Some analyses like DAPCA require low levels of missing data, so a data set was generated by filtering out all loci that were not represented in at least 20 individuals at the cost of fewer loci. On the other hand, phylogenetic analyses in RAxML are less restrictive in regards of missing data. In fact, missing data should not be removed according to Huang and Knowles (2016) because it would bias the analyses. Therefore, a large data set was generated for the phylogenetic analysis by keeping all loci that were represented by 4 or more individuals. We also generated a medium-size data set by retaining all loci that were present for at least 20 individuals, which was used for the STRUCTURE analyses and to test asymmetrical migration rates in divMigrate-online. Number of loci obtained under each minimum sample constraint is given in Table S1.

BayesAss3-SNPs was first run by using the BA3-SNPS-autotune.py script that comes with the program to fine tune the acceptance rates for proposed changes to parameters. This resuled in the following parameter values: m= 0.2125 (for the allele frequencies), a= 0.7750 (for inbreeding coefficients) , and f= 0.0375 (for migration rates). Accepted rates were as follow: migration rates= 0.45, individual migrant ancestries= 0.00, allele frequencies= 0.44, inbreeding coefficient= 0.36, and missing genotypes= 0.82. Acceptance rates are recommended to be between 20 and 60 %. Based on these results, it was not possible to get all the acceptance values between recommended range, with the individual migrant ancestries value and the missing genotypes value resulting out of range.

All data for analyses can be downloaded from: https://datadryad.org/stash/share/u30ZZTnVWdldwbDBChsJ1zuni0TVuwRFPVwcv_bQwO8

A description of each file is given below:

"Yoldias_COI_popart.nex" contains mitochondrial DNA COI sequences used to build an haplotype network in POPART (Leigh and Bryant 2015)

"Input2_NextRad_All.phy" is a concatenated data set using full-lenght reads, that includes not only the SNPs, but also the invariable sites. All loci containing data for at least 4 individuals were included in this data set. This file was used to estimate a ML tree with RAxML v.8.1.16 (Stamatakis 2014)

"Input3_NextRad_All.stru" is a Structure file created with the complete data set (41 individuals) and a minimum of 10 individuals with data for the locus to be called to the alignment which yielded nearly 8000 SNPs. This file was used to estimate population subdivision with the program STRUCTURE (Pritchard et al. 2000) and Fst values between populations (Table 2 in the main text).

"Input4_NextRad_mag.stru" is a subset of individuals used to run STRUCTURE analysis on samples from South America only. It used a minimum of 7 individuals for a locus to be included in the data set.

File "Input5_NextRad_antarctica.stru" is a subset from "branch_41_samples_yoldia_min10.u.stru" used to run STRUCTURE analysis on samples from Antarctica only. It used a minimum of 7 individuals for a locus to be included in the data set.

File "Input6_NextRad_All_genepop" is a genepop file created from file "Input3_NextRad_All.stru" and used to estimate asymmetrical gene flow among populations with the program divMigrate-Online (Sundqvist et al. 2016).

File "Input7_NextRad_All_DAPC.stru" is a .stru (STRUCTURE) input file that can be transformed using R in a genlight object to perform the discriminant analysis of principal components. This file contains 811 SNPs and was generated by filtering out all loci represented by less than 20 individuals.

File "populations_yoldias.txt" is a file containing population assignments to be used in DAPC analyses.

To run divMigrate-online it requires a genepop input file which was obtained by converting the structure file (.stru) from the STRUCTURE analysis. Firstly, the structure file is loaded into R using the command *read.structure()* from the Adegenet package. To write out the genepop file, the function *writeGenPop()* from the zvau package was used (https://rdrr.io/github/romunov/zvau/man/zvau-package.html).

**References**

Huang H, Knowles LL. 2016 Unforeseen consequences of excluding missing data from next-generation sequences: Simulation study of rad sequences. Syst. Biol. 65, 357–365. (doi:10.1093/sysbio/syu046)

**Supplementary Tables and Figures**

| Table S1: Summary statistics by sample and by population of the processing steps in iPyRAD. Last three columns show the number of loci generated under different coverage levels (minimum number of samples for a locus to be called); Min4= 4 or more samples, Min10= 10 or more samples, M20= 20 or more samples. Averages per population are given at the bottom of the table; MS= Magellan Strait, PW= Puerto Williams, BO= O’Higgins Base, Ye= Yelcho, and Ro= Rothera. | | | | | | | | | | |
| --- | --- | --- | --- | --- | --- | --- | --- | --- | --- | --- |
|  | reads_raw | reads_passed_filter | clusters_total | clusters_hidepth | hetero_est | error_est | reads_consens | Min4 | Min10 | Min20 |
| BB2_G1_3 | 1423927 | 1412486 | 160711 | 42312 | 0.0558 | 0.0168 | 23032 | 13355 | 5247 | 192 |
| EM_01 | 9420654 | 9390091 | 341064 | 134469 | 0.0399 | 0.0072 | 96770 | 10732 | 4574 | 821 |
| EM_02 | 8563470 | 8538782 | 290473 | 107469 | 0.0423 | 0.0065 | 76186 | 12248 | 4873 | 807 |
| EM_03 | 8396051 | 8370511 | 299711 | 129771 | 0.0379 | 0.0077 | 95929 | 13151 | 5320 | 787 |
| EM_04 | 8722107 | 8693594 | 343618 | 115391 | 0.0283 | 0.0046 | 90000 | 14463 | 5477 | 808 |
| EM_05 | 11630515 | 11597402 | 439317 | 140866 | 0.0308 | 0.0043 | 108308 | 11867 | 4727 | 817 |
| EM_06 | 7995902 | 7971302 | 316275 | 124947 | 0.0410 | 0.0081 | 90263 | 2337 | 1088 | 791 |
| EM_07 | 2836928 | 2819357 | 187549 | 42324 | 0.0463 | 0.0092 | 27508 | 8914 | 3740 | 271 |
| EM_08 | 6553689 | 6533757 | 324330 | 114238 | 0.0262 | 0.0051 | 91910 | 7871 | 3281 | 733 |
| PW_01 | 7478472 | 7452998 | 268367 | 98102 | 0.0405 | 0.0070 | 70315 | 7843 | 3265 | 653 |
| PW_02 | 6667109 | 6642304 | 292753 | 106646 | 0.0339 | 0.0081 | 76744 | 9669 | 4034 | 702 |
| PW_03 | 8536882 | 8511982 | 233122 | 94138 | 0.0395 | 0.0057 | 69378 | 1143 | 493 | 727 |
| PW_04 | 2022658 | 2014758 | 93440 | 22466 | 0.0504 | 0.0105 | 14078 | 7592 | 3320 | 105 |
| PW_05 | 7957774 | 7923331 | 290563 | 97720 | 0.0480 | 0.0078 | 67488 | 5503 | 2551 | 628 |
| PW_06 | 5314706 | 5292835 | 216175 | 64014 | 0.0523 | 0.0089 | 43055 | 11357 | 4743 | 532 |
| Y_Rot_1 | 5756516 | 5743479 | 332581 | 102060 | 0.0265 | 0.0047 | 81330 | 17446 | 6303 | 771 |
| Y_Rot_17 | 10241854 | 10222424 | 407385 | 145045 | 0.0264 | 0.0046 | 116842 | 11807 | 4670 | 851 |
| Y_Rot_18_wgs | 6871627 | 6858074 | 308826 | 124568 | 0.0415 | 0.0072 | 87293 | 14517 | 5736 | 751 |
| Y_Rot_19 | 8007690 | 7987681 | 322370 | 106772 | 0.0266 | 0.0042 | 86106 | 12844 | 5137 | 842 |
| Y_Rot_2 | 6597124 | 6583294 | 345319 | 107113 | 0.0264 | 0.0044 | 85402 | 12004 | 4910 | 797 |
| Y_Rot_3 | 5804152 | 5790126 | 359855 | 111093 | 0.0246 | 0.0049 | 88051 | 6399 | 2836 | 786 |
| Y_Rot_4 | 3248379 | 3240466 | 224318 | 72143 | 0.0277 | 0.0059 | 57532 | 8147 | 3650 | 524 |
| Y_Rot_7 | 3865756 | 3852992 | 303758 | 96686 | 0.0283 | 0.0063 | 74444 | 10401 | 4364 | 667 |
| Y_Rot_8 | 4860668 | 4848969 | 361283 | 109152 | 0.0271 | 0.0060 | 83198 | 1888 | 768 | 727 |
| Y_Yel_1 | 1503811 | 1490657 | 167077 | 40972 | 0.0660 | 0.0157 | 20938 | 1011 | 900 | 194 |
| Y_Yel_10 | 1073839 | 1064222 | 171782 | 33309 | 0.0401 | 0.0127 | 21422 | 3783 | 442 | 122 |
| Y_Yel_19 | 2047119 | 2034551 | 222584 | 55525 | 0.0305 | 0.0083 | 41042 | 2092 | 1836 | 411 |
| Y_Yel_2 | 1650772 | 1636585 | 224273 | 47241 | 0.0377 | 0.0096 | 32469 | 2189 | 930 | 208 |
| Y_Yel_3 | 1916523 | 1906545 | 157900 | 49031 | 0.0671 | 0.0161 | 28658 | 3253 | 963 | 188 |
| Y_Yel_4 | 1991414 | 1980348 | 174657 | 57209 | 0.0517 | 0.0120 | 34286 | 1916 | 1479 | 333 |
| Y_Yel_5 | 1614718 | 1603016 | 165595 | 44924 | 0.0575 | 0.0145 | 24653 | 2067 | 890 | 193 |
| Y_Yel_6 | 1623934 | 1611325 | 144786 | 34255 | 0.0580 | 0.0152 | 19647 | 2203 | 957 | 211 |
| Y_Yel_7 | 1673343 | 1661846 | 167550 | 44720 | 0.0557 | 0.0142 | 24434 | 1675 | 981 | 185 |
| Ye_BO_34 | 5007425 | 4990198 | 293990 | 107377 | 0.0345 | 0.0090 | 77310 | 6737 | 2854 | 596 |
| Ye_BO_35 | 3222550 | 3206748 | 290739 | 79024 | 0.0305 | 0.0079 | 59894 | 4658 | 2072 | 442 |
| Ye_BO_36 | 7067018 | 7038006 | 313943 | 113612 | 0.0423 | 0.0083 | 80065 | 8883 | 3787 | 702 |
| Ye_BO_37 | 2962253 | 2944687 | 244643 | 71633 | 0.0464 | 0.0132 | 46040 | 3657 | 1659 | 409 |
| Ye_BO_38 | 4280765 | 4267541 | 259193 | 108299 | 0.0470 | 0.0109 | 73934 | 6561 | 2622 | 515 |
| Ye_BO_39 | 1997602 | 1985813 | 161215 | 51660 | 0.0585 | 0.0163 | 31062 | 2449 | 1036 | 243 |
| Ye_BO_40 | 3468789 | 3452213 | 375341 | 113859 | 0.0307 | 0.0096 | 83408 | 4273 | 1637 | 356 |
| Ye_BO_41 | 1747298 | 1735659 | 189720 | 44161 | 0.0371 | 0.0117 | 31229 | 2397 | 1086 | 257 |
| Total Average | 4,966,434 | 4,948,853 | 263,126 | 85,520 | 0.0405 | 0.0090 | 61,748 | 7,154 | 2,957 | 528 |
|  |  |  |  |  |  |  |  |  |  |  |
| **Summaries**  **by Population** |  |  |  |  |  |  |  |  |  |  |
| MS | 8,014,915 | 7,989,350 | 317,792 | 113,684 | 0.0366 | 0.0066 | 84,609 | 10,198 | 4,135 | 729 |
| PW | 6,329,600 | 6,306,368 | 232,403 | 80,514 | 0.0441 | 0.0080 | 56,843 | 7,185 | 3,068 | 558 |
| OB | 3,719,213 | 3,702,608 | 266,098 | 86,203 | 0.0409 | 0.0109 | 60,368 | 4,952 | 2,094 | 440 |
| Ye | 1,651,940 | 1,640,158 | 175,692 | 44,950 | 0.0520 | 0.0135 | 27,058 | 3,354 | 1,463 | 224 |
| Ro | 6,139,307 | 6,125,278 | 329,522 | 108,292 | 0.0283 | 0.0054 | 84,466 | 10,606 | 4,264 | 746 |

Table S2: *Fst* values (above the diagonal) and p-values (below the diagonal) between populations of *Aequiyoldia eightsi* using the mtDNA (COI) data. P-values were obtained based on Monte-Carlo tests with 999 permutations. Fst values in bold are statistically significant.

|  | Puerto Williams (PW) | Maguellan Strait (MS) | O'Higgins Base (BO) | Yelcho (Yel) | Rothera (Rot) |
| --- | --- | --- | --- | --- | --- |
| PW | - | 0.142 | **0.930** | **0.944** | **0.965** |
| MS | 0.131 | - | **0.956** | **0.967** | **0.986** |
| BO | 0.001 | 0.001 | - | **0.360** | **0.469** |
| Yel | 0.001 | 0.001 | 0.012 | - | 0.072 |
| Rot | 0.001 | 0.001 | 0.008 | 0.304 | - |

Table S3: Asymmetrical migration rates calculated with divMigrate-online using Jost’s *D* distances. Values in bold are statistically significant based on 200 bootstrap replicates.

| Sites | Magellan Strait | Puerto Williams | O’Higgins Base | Yelcho | Rothera |
| --- | --- | --- | --- | --- | --- |
| Magellan Strait | - | 0.803 | 0.052 | 0.04 | **0.094** |
| Puerto Williams | 1 | - | **0.048** | 0.038 | **0.085** |
| O’Higgins Base | 0.053 | 0.044 | - | 0.15 | **0.693** |
| Yelcho | **0.042** | 0.038 | **0.166** | - | **0.295** |
| Rothera | 0.064 | 0.055 | 0.4 | 0.173 | - |

Table S4: BayesAss migration estimates (as proportion of migrant individuals in population). Migration rates represent migration from the population on the top into the population on the left.

|  | Magellan Strait | Puerto Williams | O’Higgins | Yelcho | Rothera |
| --- | --- | --- | --- | --- | --- |
| Magellan Strait | 0.8718(0.0435) | 0.0261(0.0248) | 0.0258(0.0241) | 0.0509(0.0318) | 0.0255(0.0235) |
| Puerto Williams | 0.0298(0.0273) | 0.8490(0.0480) | 0.0301(0.0274) | 0.0612(0.0374) | 0.0299(0.0276) |
| O’Higgins | 0.0253(0.0232) | 0.0256(0.0234) | 0.8970(0.0412) | 0.0260(0.0244) | 0.0262(0.0238) |
| Yelcho | 0.0223(0.0206) | 0.0220(0.0209) | 0.0221(0.0209) | 0.9117(0.0366) | 0.0219(0.0203) |
| Rothera | 0.0235(0.0219) | 0.0236(0.0220) | 0.0235(0.0218) | 0.0236(0.0220) | 0.9057(0.0387) |

Supplementary Figures


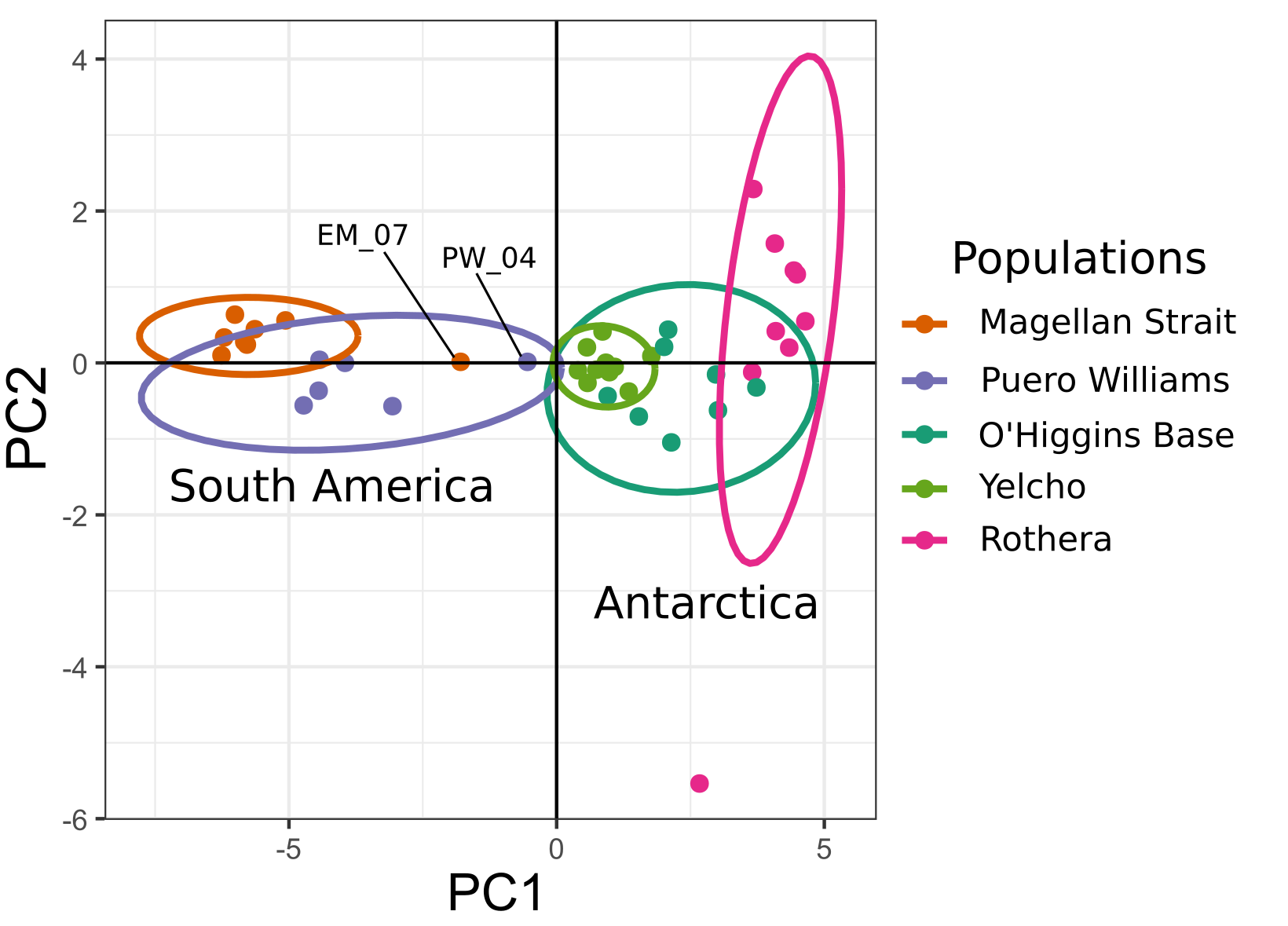


Figure S1: Principal component analysis for 41 samples from South America and Antarctica based on a matrix of 811 SNPs (with a minimum of 20 individuals with data per loci). Labels pointing to dots indicate those two samples that had the fewest loci in the genomic data set. Note the proximity of these dots to the center of the plot (0,0) that makes them look closer to the Antarctic site Yelcho, likely an artefact given the low information available (high missing data) for these samples.


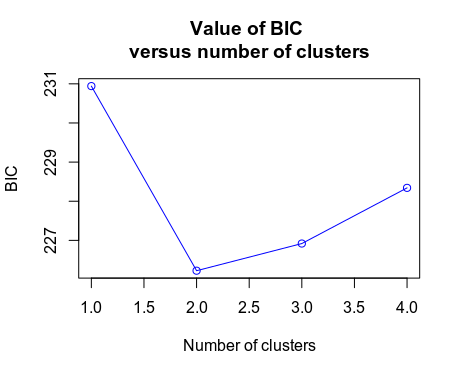


Figure S2: This graph shows BIC scores for different valus of k. It is observed a decrease until k = 2 clusters, after which BIC increases. In this case, the elbow in the curve matches the smallest BIC, and indicates 2 clusters should be retained.


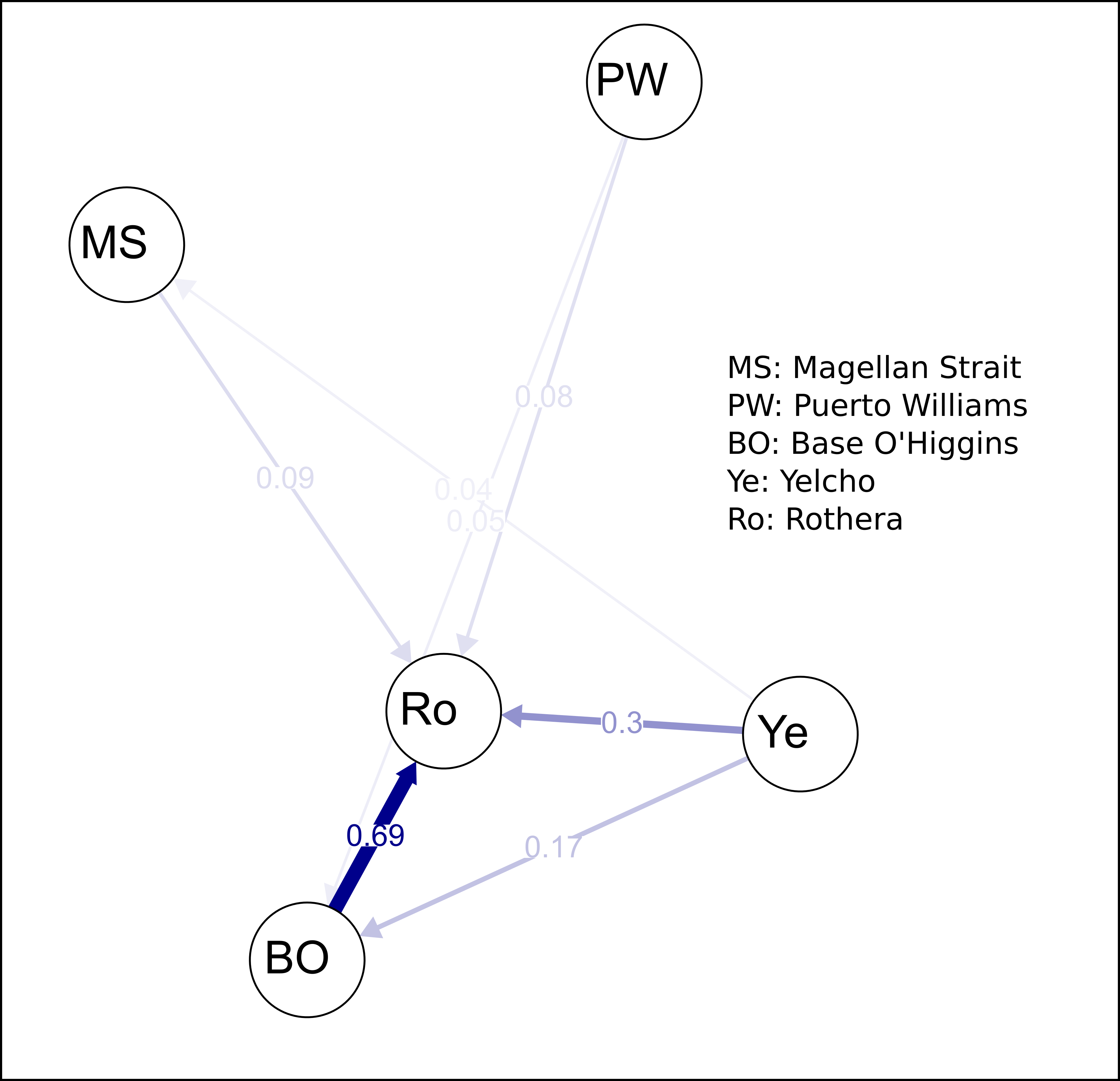


Figure S3: Asymmetrical migration rates between five populations of *Aequiyoldia eightsii* from South America and the West Antarctic Peninsula calculated with divMigrate -online based on Jost’s *D.* Thicker arrows represent higher migration rates. The input file “Input6_NextRad_All_genepop” was used for this analysis, and consisted of 41 individuals and 7973 loci, with a minimum of 10 individuals with data per loci.


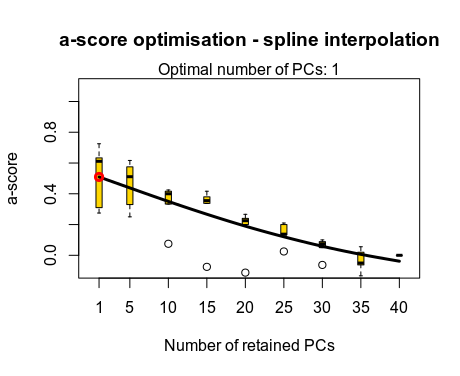


Figure S6: Results of a-score as a function of the number of retained PC axes. The plot suggest the retention of 1 PC to conduct the DAPC analysis.


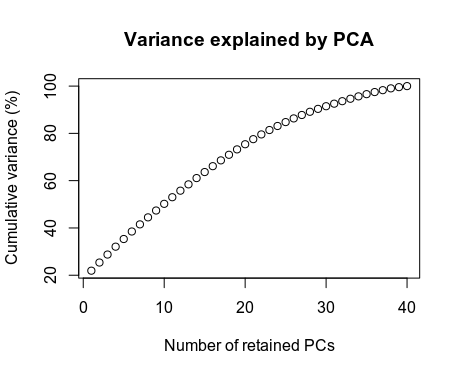


Figure S7: Cumulative variance explained by retained PC axes.


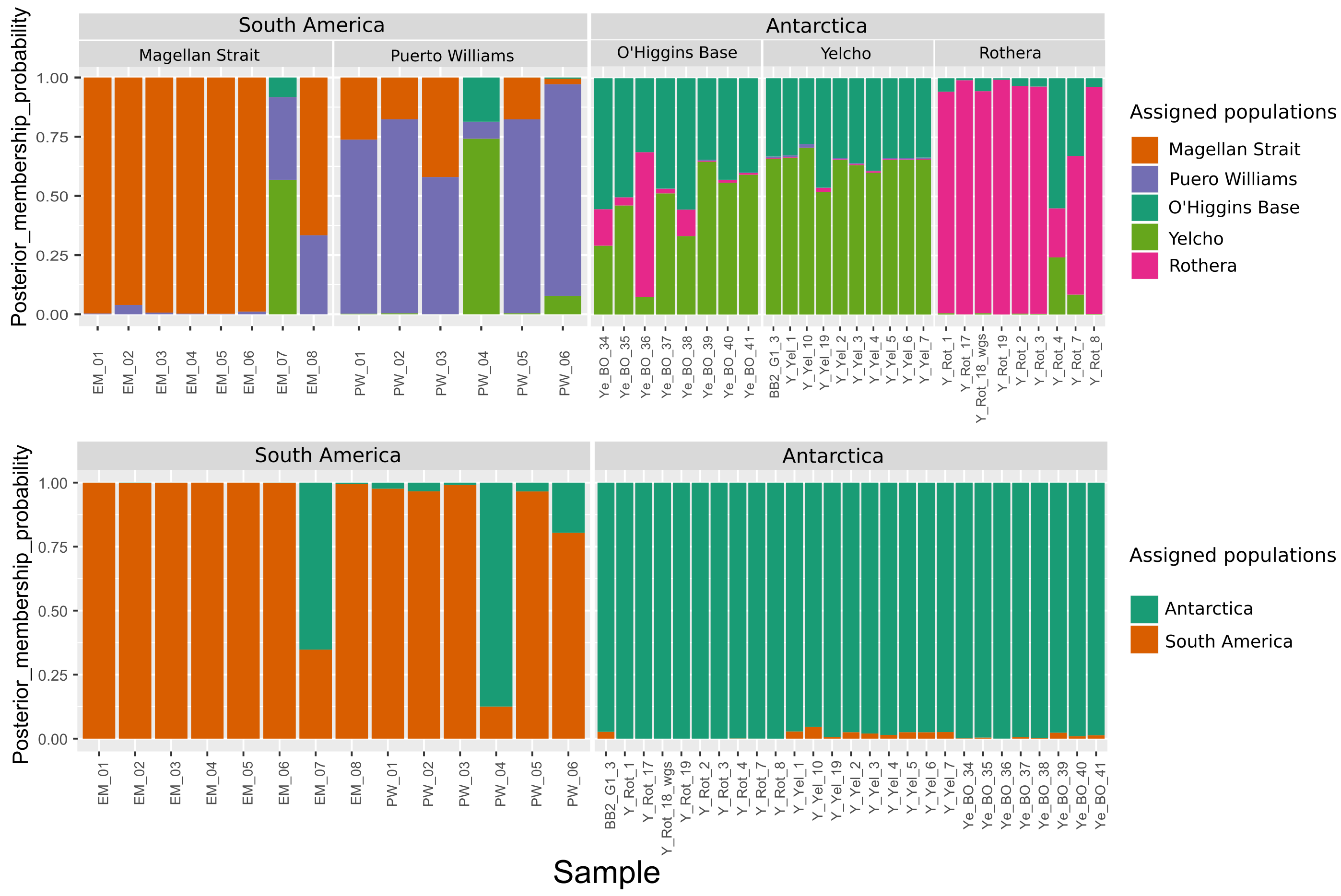


Figure S8: Membership probabilities derived from the discriminant analyses of principal components. The upper plot shows memberships for k= 5, whereas the lower plot shows memberships for k= 2.
